# Dietary Supplements/Antioxidants: Impact on Redox Status in Brain Diseases

**DOI:** 10.1155/2017/5048432

**Published:** 2017-04-26

**Authors:** Gilles J. Guillemin, Musthafa Mohamed Essa, Byoung-Joon Song, Thamilarasan Manivasagam

**Affiliations:** ^1^Neuroinflammation Group, Department of Biomedical Research, Faculty of Medicine and Health Sciences, Macquarie University, Sydney, NSW, Australia; ^2^Department of Food Science and Nutrition, CAMS, Sultan Qaboos University, Muscat, Oman; ^3^Laboratory of Metabolism and Membrane Biochemistry, National Institute on Alcohol Abuse and Alcoholism, National Institutes of Health, Bethesda, MD, USA; ^4^Department of Biochemistry and Biotechnology, Faculty of Science, Annamalai University, Annamalainagar, Tamil Nadu, India

While the saying “wisdom and grace come with age” may be true, unfortunately, ageing also comes with a package of decreased mental abilities and an increased risk of dementia. As the world's population continues to age, Alzheimer's disease and dementia have become the largest causes of death in developed countries. Maintaining good brain health has become a major priority, and, over several decades, scientists have identified some simple factors that can reduce this cognitive decline, including regular exercise and/or a healthy diet.

The brain uses roughly 20% of all the body oxygen intake making neurons particularly susceptible to oxidative and free radical damage. Oxidative stress, or “brain rust,” is one of the deleterious processes that progressively increases during the ageing process. It leads to cognitive decline and often to neurodegenerative diseases. There are two main sources for reactive oxygen species (ROS) within the central nervous system: (1) mitochondria are producing ROS as a result of the normal production of energy in all cells and (2) activated microglia (the immune cells of the brain) are also producing ROS as part of the “weaponry” they use to destroy pathogens or attack abnormal proteins accumulating in the brain.

Under physiological conditions, there is an equilibrium between ROS production and catabolism in the body. It is important to highlight that ROS are essential for cells to function normally. The lack of ROS production would alter the level of energy production in the body and especially the brain. In ageing or neuroinflammatory conditions, there is a significant increase in oxidative stress and ROS production which damages DNA, lipids of cell membranes, and proteins. Within the brain, this oxidative state will lead to progressive neuronal damage and loss of normal cerebral functions. However, the human body has several endogenous antioxidant defence mechanisms to protect cells from the damage associated with oxidative stress. These mechanisms include several antioxidant enzymes, such as catalase, superoxide dismutases, superoxide reductases, and glutathione peroxidases.

Another alternative to decrease oxidative stress is to take processed or synthetic antioxidants with the aim of slowing down the ageing process, and extending life span, as promised by many brands. Antioxidant supplements represent a market of more than $60 billion worldwide per year with the USA alone accounting for $7 billion of market share.

A better, and far less expensive option is to get these antioxidants from a healthy diet as many plants and fruits contain potent natural antioxidant compounds that can protect cells against oxidative stress [1]. Most of these compounds are able to cross the blood-brain barrier and neutralize free radicals in the brain [2, 3], even if some of them need to be processed by gut bacteria first. The active compounds of curcumin and pomegranate [4–6] are amongst these.

The use of natural antioxidants against free radical toxic conditions is an emerging field in the management of age-related illnesses and neurodegenerative diseases [7]. These antioxidants could offer neuroprotective, neurotropic, and proneurogenic support to overcome the motor and cognitive impairments which occur during these age-related diseases [4, 5]. This special issue provides new scientific insights and reviews on the ability of various antioxidants to protect the brain during age-related diseases such as Alzheimer's, Parkinson's and Huntington's. Other articles focus on the importance of plants such as curcumin, lemon balm, and rosemary for the treatment of brain tumours A study describes the ability of 3,5-dicaffeoylquinic acid, a polyphenol compound found in edulis morning glory, to improve learning and memory deficits. Finally, a publication describes the neuroprotective effects of Brazilian green propolis, a compound collected and produced by bees.

This special issue highlights the importance of some specific natural molecules in human health. As an outcome, the natural products and their active materials discussed in this issue could provide a novel lead for therapeutic strategies for ageing and neurodegenerative conditions.

*Gilles J. Guillemin* *Musthafa Mohamed Essa* *Byoung-Joon Song* *Thamilarasan Manivasagam*
